# First human isolation of *Gibbsiella quercinecans* associated with infection - significance and relevance

**DOI:** 10.1016/j.nmni.2026.101867

**Published:** 2026-10-08

**Authors:** Jonas Lübbe, Leonie Schaudig, Jiabin Huang, Konstantin Tanida, Martin Christner, Martin Aepfelbacher, Susanne Rein, Holger Rohde, Benjamin Berinson

**Affiliations:** a Institute of Medical Microbiology, Virology and Hygiene, University Medical Center Hamburg-Eppendorf (UKE), Hamburg, Germany; b Department for Hand-, Plastic- and Microsurgery, Burn Unit, Berufsgenossenschaftliches Klinikum, Hamburg, Germany; c Department of Trauma and Orthopedic Surgery, University Medical Center Hamburg-Eppendorf (UKE), Hamburg, Germany

**Keywords:** *Gibbsiella* spp., Acute oak decline, Plant pathogen, NGS, 16S rDNA, ONT, Skin infection, Soft tissue infection, Case report

## Abstract

*Gibbsiella quercinecans* is a plant-associated environmental bacterium described in the context of Acute Oak Decline and has not previously been reported in human clinical samples.

A previously healthy 60-year-old male developed an outdoor trauma-associated skin and soft tissue infection of a primarily sutured left-thumb wound. Surgical exploration was performed and an intraoperative specimen of representative infected tissue from the depth of the wound yielded a Gram-negative rod in pure culture. MALDI-TOF MS, automated biochemical testing, and 16S rDNA sequencing were inconclusive for species-level identification. Definitive identification as *G. quercinecans* was achieved by whole-genome sequencing. The isolate was susceptible to all tested antimicrobial agents and no known relevant acquired resistance genes were detected. The infection subsided after surgical debridement, irrigation, and antimicrobial therapy.

This case represents, to our knowledge, the first isolation of *G. quercinecans* from a human infection and highlights the value of genomic methods for identifying unusual environmental pathogens at the interface of outdoor exposure and clinical microbiology.

## 1 Introduction

*Gibbsiella quercinecans* was first described in 2010 in association with Acute Oak Decline in the United Kingdom and Spain and is regarded as a plant-associated environmental bacterium implicated in tree diseases [1]. Other members of the genus *Gibbsiella* have been isolated from environmental and animal sources, including the intestinal tract of butterflies and the oral cavity of bears [2,3]. Here, we report, to our knowledge, the first human isolation of *G. quercinecans*, recovered from a deep intraoperative specimen obtained from a patient with phlegmonous soft tissue infection following outdoor trauma. The identification in human-derived material constitutes an unexpected and significant observation, warranting further investigation into its clinical relevance.

## 2 Case presentation

A 60-year-old male with no relevant past medical history presented to the emergency department after a bicycle-related fall onto paving stones in Northern Germany, resulting in lacerations of the scalp and left thumb. The accident occurred in close proximity to oak trees (*Quercus robur*). Both wounds appeared superficial at initial assessment, were debrided, irrigated and sutured, and the patient was discharged in stable condition.

Two days later, he presented to his general practitioner with increasing pain, erythema, and purulent secretion of the wound of the left thumb, while the scalp wound remained without signs of infection. He remained afebrile.

Due to progressive local inflammation, he was referred to the hospital, where surgical exploration of the left thumb was indicated. Preoperative laboratory findings showed a C-reactive protein level of 16.2 mg/L and a leukocyte count of 7.77 × 10^9^/L, indicating a mild inflammatory response without leukocytosis. No bony lesion or signs of osteitis were detected on x-rays of the thumb taken preoperatively in two planes. Intraoperatively, purulent wound exudate was encountered, accompanied by localized extensor tendon synovitis. At the level of the interphalangeal joint, the long extensor tendon was partially transsected with approximately 30% of its diameter, and the joint capsule was lesioned resulting in exposure of the joint cartilage. The joint contained clear synovial fluid, and the articular cartilage appeared intact; there was no clinical evidence of a joint empyema.

Representative samples of infected subcutaneous and tenosynovial tissue were obtained under sterile conditions for microbiological analysis. Radical debridement of all infected tissue was performed, followed by irrigation of the joint with diluted gentamicin solution. After further irrigation, the long extensor tendon of the thumb was reconstructed with appropriate suture technique and the wound was subsequently closed.

The patient was hospitalized for five days and received empiric intravenous cefazolin. Inflammatory markers declined, and local findings improved. Postoperatively, reduced tendon function was noted, and physiotherapy was initiated. Upon discharge, oral antimicrobial therapy with amoxicillin/clavulanic acid was continued for additional 5 days.

### 2.1 Microbiology

The specimen was processed according to routine microbiological procedures and inoculated onto chocolate agar and Columbia agar with 5% sheep blood supplemented with colistin and aztreonam followed by aerobic incubation at 37 °C in 5% CO_2_. Anaerobic culture was performed on Schaedler agar (media: Oxoid, Basingstoke, UK and Thermo Fisher Scientific, Waltham, MA, USA). Plates were examined after 24 and 48 h; anaerobic cultures were evaluated after two and seven days.

After 24 h of incubation, medium-sized, grey, smooth, moist, non-haemolytic colonies with entire margins were observed on chocolate agar. No additional aerobic or anaerobic bacterial growth was detected. Further characterization showed colourless colonies on MacConkey agar consistent with a non–lactose fermenting phenotype. Growth was also observed at room temperature and at 27 °C.

Initial identification was attempted using matrix-assisted laser desorption ionization time-of-flight mass spectrometry (MALDI-TOF MS; Bruker, Bremen, Germany) employing the MBT Compass software with the HAT IVD Library (IVD-12299 Library module, 2023 version). Identification remained inconclusive despite adequate spectral quality, as it ultimately turned out, that the organism was not represented in the reference database. The closest matches included various *Serratia* spp.; however, all identification scores remained below the threshold for reliable genus- or species-level identification (<1.7). Automated biochemical identification using VITEK® 2 GN ID cards (bioMérieux, Marcy-l'Étoile, France) misidentified the isolate as *Serratia plymuthica* with a probability of 88 to 92%, despite discordant phenotypic characteristics.

Partial 16S rDNA gene sequencing indicated affiliation with the genus *Gibbsiella,* but did not allow definitive species-level assignment. For definitive species-level identification, whole-genome sequencing (WGS) was conducted using the Oxford Nanopore Technologies (ONT) platform, generating high-quality long-read sequences. The genome was assembled with Unicycler v0.5.0 [4], resulting in a complete, circular chromosome of 5,672,081 base pairs. No plasmids were detected. OrthoANIu v1.2 [5] analysis of the newly assembled genome against the genomes of *Gibbsiella quercinecans* strains FRB97 and DSM 25889 (GenBank: NZ_CP014136.1 and NZ_SMBC01000001.1) revealed an average nucleotide identity (ANI) of 98.32% for both, well above the species threshold (∼95–96%) [6,7]. Phylogenetic analysis demonstrated that the isolate clustered with *G. quercinecans* reference strains and was clearly separated from other *Gibbsiella* species and the biochemically similar genus *Serratia* (Fig. 1), thereby confirming species-level classification.

**Fig. 1:**
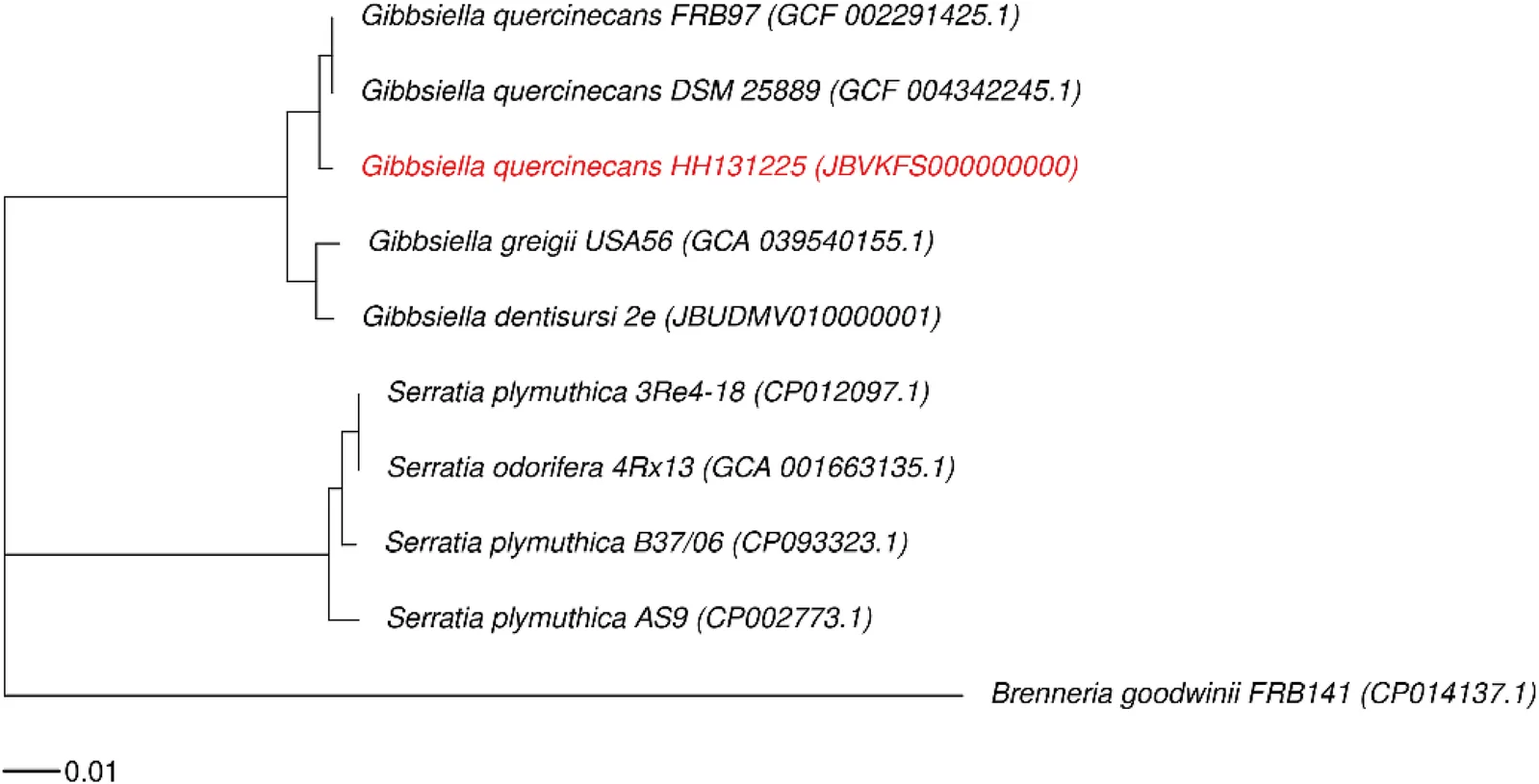
Core-genome maximum-likelihood phylogeny of *Gibbsiella quercinecans* HH131225 (red) with reference strains from the genus *Gibbsiella*, biochemically similar *Serratia* spp., and *Brenneria* outgroup. Tip labels show species, strain designation, and GenBank assembly accession or chromosome accession. Scale bar: substitutions per site. Tree inferred from 60,736 core-genome SNPs using Roary [8] and RAxML-NG [9] with the GTR + G model and 1000 bootstrap replicates.

Antimicrobial resistance (AMR) gene profiling was conducted using ABRicate v1.0.1 (https://github.com/tseemann/abricate), referencing the MEGARes v3.00 [10] and CARD v3.2.8 [11] databases. Three genes associated with efflux were identified: *CRP*, a global regulator that represses MdtEF expression; and two RND efflux pumps (*SDEX* and *SDEY*), linked to multi-compound drug and biocide resistance. No known acquired antimicrobial resistance determinants were identified beyond these hits. Virulence factor analysis using VFDB v2026-Feb-06 [12] did not identify any matches to known virulence-associated genes. Default parameters were used for all tools unless stated otherwise.

Antimicrobial susceptibility testing was performed using disc diffusion and VITEK® 2 according to EUCAST breakpoints for Enterobacterales (version 16). The isolate was susceptible to ampicillin, ampicillin/sulbactam, piperacillin/tazobactam, cefotaxime, ceftazidime, ertapenem, imipenem, meropenem, ciprofloxacin and trimethoprim/sulfamethoxazole. The minimum inhibitory concentration for ampicillin, also verified with gradient diffusion, was close to the clinical susceptibility breakpoint, but remained within the susceptible range (8 mg/L). MICs for selected agents determined by Etest (bioMérieux, Marcy-l’Etoile, France; Liofilchem, Roseto degli Abruzzi, Italy) are shown in Table 1.

**Table 1:** MICs determined by gradient diffusion (Etest) and EUCAST interpretation (Enterobacterales, version 16).

Antibiotic (Etest)	MIC (mg/L)	Interpretation (EUCAST v16)
ampicillin	8	S
ampicillin/sulbactam	0.25	S
piperacillin/tazobactam	1	S
cefotaxime	0.032	S
meropenem	0.016	S
ciprofloxacin	0.004	S
trimethoprim/sulfamethoxazole	0.064	S

S: susceptible.

## 3 Discussion and conclusions

Primary skin and soft tissue infections (SSTIs) are mainly attributed to a limited number of well-characterized bacterial pathogens [13]. In contrast, secondary SSTIs, particularly those following trauma, often present as polymicrobial infections involving aerobic and anaerobic organisms, including environmental bacteria [14]. In the present case, *Gibbsiella quercinecans* was isolated from a deep intraoperative specimen.

The clinical presentation was consistent with a localized infection, with increasing pain, erythema, and purulent exudate. Recovery of the organism in pure culture from a deep surgical specimen, in the absence of alternative pathogens, together with the favourable response to surgical and antimicrobial therapy, supports an etiological role. To our knowledge, this is the first report of *G. quercinecans*—and, in fact, of any member of the genus *Gibbsiella*—associated with a human infection.

Beyond its original description as one of the bacterial species associated with Acute Oak Decline [1], *G. quercinecans* has since been detected in additional geographic and ecological settings, including reports from Poland [15] and association with bacterial canker of Russian olive [16]. These findings suggest a broader ecological and geographical distribution than initially appreciated. It is conceivable that environmental and climate-related stressors may influence the distribution of Acute Oak Decline and its associated bacterial communities. Whether this could result in increased human exposure to *G. quercinecans* remains unknown. The organism has furthermore been shown to persist in environmental substrates such as forest soil and rainwater [17], supporting the plausibility of environmental exposure in the present case, in which the injury occurred outdoors in close proximity to oak trees (*Quercus robur*). In plant disease settings, the organism is typically detected as part of polymicrobial communities rather than as a sole pathogen [18]. In contrast, the present case yielded monomicrobial growth from a deep surgical specimen. Environmental inoculation at the time of trauma, facilitated by disruption of skin integrity, therefore appears biologically plausible. Primary wound closure may have hindered local clearance of inoculated microorganisms. *G. quercinecans* is capable of growth across a relatively broad temperature range, including at 37 °C, as also observed for the present isolate. This temperature tolerance is compatible with both environmental persistence and growth in the human host. This interpretation is further supported by occasional trauma-associated infections caused by other plant-associated Enterobacterales such as *Pantoea agglomerans* [19] and *Kosakonia cowanii* [20,21].

Whole-genome analysis did not identify known acquired antimicrobial resistance determinants represented in the applied reference databases. Database hits were limited to chromosomally encoded efflux-associated elements (*CRP*, *SDEX*, and *SDEY*), consistent with the largely susceptible phenotype. Because this analysis is limited to resistance determinants represented in current reference databases, novel or uncharacterized resistance mechanisms in *G. quercinecans* cannot be excluded. Similarly, no matches to known virulence-associated genes were identified in VFDB, although uncharacterized virulence determinants may be present. Further limitations of the here presented case are the absence of molecular confirmation directly from the primary specimen and the lack of histopathological correlation since no sample was sent for workup to the pathology.

The diagnostic work-up illustrates limitations of conventional identification methods. MALDI-TOF MS and automated biochemical systems did not allow reliable species-level identification and instead suggested *Serratia* spp. with low confidence scores. Definitive identification was achieved only through molecular analysis. These findings emphasize the value of genomic approaches for accurate characterization of unusual clinical isolates and indicate that environmental organisms may be underrecognized and can occur even in immunocompetent individuals following trauma.

## CRediT authorship contribution statement

**Jonas Lübbe:** Writing – original draft, Methodology, Investigation, Formal analysis, Data curation. **Leonie Schaudig:** Writing – review & editing, Investigation, Data curation. **Jiabin Huang:** Writing – review & editing, Formal analysis, Data curation. **Konstantin Tanida:** Writing – review & editing, Formal analysis. **Martin Christner:** Writing – review & editing, Methodology, Investigation. **Martin Aepfelbacher:** Writing – review & editing, Supervision, Resources. **Susanne Rein:** Writing – review & editing, Investigation, Data curation. **Holger Rohde:** Writing – review & editing, Supervision. **Benjamin Berinson:** Writing – original draft, Supervision, Project administration, Conceptualization.

## Availability of data and materials

The isolate has been deposited at the German Collection of Microorganisms and Cell Cultures (DSMZ) under submission number DSM 121826.

The whole-genome sequencing project for *Gibbsiella quercinecans* strain HH131225 has been deposited at NCBI under BioProject accession PRJNA1424548 and BioSample accession SAMN55386717. The raw sequencing reads are available in the Sequence Read Archive (SRA) under accession SRR37253647. The complete, circular genome sequence has been submitted to GenBank under accession JBVKFS000000000.

## Consent for publication

Written informed consent for publication of clinical details was obtained from the patient.

## Declaration of generative AI use

During the preparation of this manuscript, the authors used ChatGPT (OpenAI, San Francisco, CA, USA) to assist with language editing, restructuring of sentences, and refinement of wording. The authors critically reviewed and revised all content and take full responsibility for the final manuscript.

## Funding

The data was gathered during routine diagnostic and the study was financed from intramural funds.

## Declaration of competing interest

The authors declare that they have no known competing financial interests or personal relationships that could have appeared to influence the work reported in this paper.

## Abbreviations

**AMR**: antimicrobial resistance
**ANI**: average nucleotide identity
**CARD**: Comprehensive Antibiotic Resistance Database
**DSMZ**: German Collection of Microorganisms and Cell Cultures (Deutsche Sammlung von Mikroorganismen und Zellkulturen)
**EUCAST**: European Committee on Antimicrobial Susceptibility Testing
**MALDI-TOF MS**: matrix-assisted laser desorption ionization time-of-flight mass spectrometry
**MIC**: minimum inhibitory concentration
**NGS**: next-generation sequencing
**ONT**: Oxford Nanopore Technologies
**SRA**: Sequence Read Archive
**SSTIs**: skin and soft tissue infections
**VFDB**: Virulence Factor Database
**WGS**: whole-genome sequencing
**16S rDNA**: 16S ribosomal DNA
